# Semiparametric methods for genome-wide linkage analysis of human gene expression data

**DOI:** 10.1186/1753-6561-1-s1-s83

**Published:** 2007-12-18

**Authors:** Guoqing Diao, DY Lin

**Affiliations:** 1 Department of Statistics, George Mason University, 4400 University Drive, MS 4A7, Fairfax, Virginia 22030, USA; 2 Department of Biostatistics, University of North Carolina, McGavran-Greenberg Hall, CB #7420, Chapel Hill, North Carolina 27599, USA

## Abstract

With the availability of high-throughput microarray technologies, investigators can simultaneously measure the expression levels of many thousands of genes in a short period. Although there are rich statistical methods for analyzing microarray data in the literature, limited work has been done in mapping expression quantitative trait loci (eQTL) that influence the variation in levels of gene expression. Most existing eQTL mapping methods assume that the expression phenotypes follow a normal distribution and violation of the normality assumption may lead to inflated type I error and reduced power. QTL analysis of expression data involves the mapping of many expression phenotypes at thousands or hundreds of thousands of marker loci across the whole genome. An appropriate procedure to adjust for multiple testing is essential for guarding against an abundance of false positive results. In this study, we applied a semiparametric quantitative trait loci (SQTL) mapping method to human gene expression data. The SQTL mapping method is rank-based and therefore robust to non-normality and outliers. Furthermore, we apply an efficient Monte Carlo procedure to account for multiple testing and assess the genome-wide significance level. Particularly, we apply the SQTL mapping method and the Monte-Carlo approach to the gene expression data provided by Genetic Analysis Workshop 15.

## Background

With the availability of high-throughput microarray technologies to measure the expression levels of many thousands of genes simultaneously, investigators have developed a vast amount of statistical and computational methods for analyzing microarray data in the last decade. On the other hand, because of the abundance of single-nucleotide polymorphisms (SNPs) as well as the modern genotyping technologies, tremendous efforts have been focused on the genetic mapping of complex human diseases, many of which are associated with quantitative traits. However, limited work has been done in combining gene expression data and marker genotype data and detecting expression quantitative trait loci that influence the variation in levels of gene expression.

There are important challenges in mapping of eQTL. First, it is well known that microarray data are noisy due to systematic biases and appropriate normalization procedures are needed to adjust for such biases. Many expression phenotypes may be non-normally distributed even after proper normalization procedures, as is evident by the gene expression data provided by Genetic Analysis Workshop 15 (GAW15). Commonly used eQTL mapping methods such as the standard variance-component (VC) approach implemented in programs SOLAR [1] and Merlin [2] assume that the expression phenotypes follow a normal distribution. However, violation of the normality assumption may lead to inflated type I error and reduced power. The other challenge is the multiple testing introduced in the mapping of many expression phenotypes at thousands or hundreds of thousands of marker loci across the whole genome. A proper procedure to adjust for multiple testing is essential for guarding against an abundance of false-positive results.

To overcome the aforementioned challenges, we first applied the semiparametric quantitative trait loci mapping method of Diao and Lin [3] to gene expression data. The SQTL mapping method is rank-based and therefore less sensitive to non-normality and outliers. Next, we used an efficient Monte Carlo procedure to assess the genome-wide significance level. The usefulness of the SQTL method and the Monte-Carlo approach is demonstrated through an application to the gene expression data provided by GAW15, which were previously analyzed by Morley et al. [4].

## Methods

### Semiparametric QTL mapping

Very recently, Diao and Lin [3] proposed the so-called SQTL mapping method for human pedigrees by allowing a completely unspecified transformation on trait values. Specifically, the phenotypic variation after the unspecified transformation is partitioned into fixed effects due to environmental variables and random effects due to major gene, polygene, and residual errors. In the context of VC analysis, the SQTL tends to be more powerful than the regression-based methods, including the one used in Morley et al. [4], as is shown in the simulation studies in Diao and Lin [3]. Moreover, the SQTL approach is rank-based and less sensitive to non-normality and outliers. Simulation studies in Diao and Lin [3] demonstrate that the SQTL is as powerful as the standard VC method assuming normality and tends to be more powerful than the standard VC method when the phenotype data are non-normally distributed or there exist outliers.

For the gene expression data, we repeatedly applied the SQTL and perform a genome-wide linkage scan for each expression phenotype. The resultant likelihood ratio test statistics or LOD scores for testing *H*_0 _: $\sigma_{g}^{2}$ = 0 vs. *H*_*A *_: $\sigma_{g}^{2}$ > 0 can be stored in a matrix with the (*i*, *j*)^th ^component corresponding to the result for mapping the *i*^th ^expression phenotype at the *j*^th ^marker on the genome, where $\sigma_{g}^{2}$ is the variance component attributable to the major gene locus. Under the null hypothesis of no linkage at a marker locus, the likelihood-ratio test statistic has an approximate distribution of $\frac{1}{2}\chi_{0}^{2}:\frac{1}{2}\chi_{1}^{2}$.

### Adjustment for multiple testing

In eQTL mapping, one performs testing on thousands of marker loci for each expression trait and one thus needs to adjust for multiple testing to guard against an abundance of false-positive results. Carlborg et al. [5] suggested that significance testing should be based on empirical genome-wide significance thresholds that are derived for each trait separately. Lin and Zou [6] and Lin [7] proposed an efficient Monte Carlo approach to adjusting for multiple comparisons in a genomic study. The Monte Carlo approach involves repeatedly generating standard normal random variables to approximate the joint distribution of the test statistics under the null distribution. Unlike the permutation resampling approach, the Monte Carlo approach does not involve repeated analyses of simulated data sets and is thus computationally less demanding. Computing is a critical issue in practice because one may perform millions of tests for each gene expression trait. For example, to perform genome-wide linkage scans for all expression phenotypes in the GAW15 (Problem 1) data set, one needs to perform approximately 10.2 million tests. Even if we only perform 1000 permutation tests for each phenotype, the total number of tests increases to 10.2 billion, which makes it almost impossible with current computing power. To obtain results at more stringent genome-wide significance levels, even more permutations are required. Furthermore, the Monte Carlo approach is widely applicable in practical situations, whereas permutation tests may not always be applicable in the presence of covariates and nuisance parameters, especially when the covariates are related to the pedigree structure such as age and gender. For sibship data, one can simply decouple the marker data from the phenotype data, with any covariates following the phenotype data in permutation tests. This strategy, however, may not always work for pedigree data. For example, if covariates include age and gender, the permutation of the covariates together with phenotype data may not be consistent with the pedigree structure because age and gender are not interchangeable between parents and offspring.

To apply the Monte Carlo approach to the gene expression data, we assess the genome-wide significance level for each expression trait and obtain the trait-specific adjusted *p*-values as described in Lin [7]. Note that the test in SQTL is one-sided and corresponding adjustment is required [6].

## Results

The gene expression data provided to GAW15 include 14 three-generation CEPH (Centre d'Etude du Polymorphisme Humain) Utah families, each of which consists of 13 or 14 individuals. We performed genome-wide linkage scans for expression levels of 3554 genes at 2882 SNPs using SQTL. We utilized SOLAR to estimate the identity-by-descent (IBD) allele-sharing probabilities at each SNP locus after eliminating Mendelian inconsistencies. Gender is included in the model as a covariate. The genome-wide significance levels and the adjusted *p*-values for each trait were based on 100,000 Monte Carlo samples. The average thresholds (on a LOD scale) at the genome-wide significance levels of 0.05, 0.01, 1.0 × 10^-3^, 1.0 × 10^-4^, and 1.0 × 10^-5 ^are 1.34, 1.70, 2.50, 3.59, and 4.68 corresponding to point-wise *p*-values of 6.5 × 10^-3^, 2.6 × 10^-3^, 3.5 × 10^-4^, 2.4 × 10^-5^, and 1.7 × 10^-6^, respectively. The relatively low thresholds compared to those used in Morley et al. [4] may reflect the strong correlations among the test statistics across the genome.

At the genome-wide significance level of 0.05, for 954 expression phenotypes, there was evidence of linkage to specific chromosomal regions. We found 356, 200, 185, and 179 expression phenotypes with evidence of linkage at the genome-wide significance levels of 0.01, 1.0 × 10^-3^, 1.0 × 10^-4 ^and 1.0 × 10^-5^, respectively. We detected 58 more expression traits at the genome-wide significance level of 1.0 × 10^-3 ^than Morley et al. [4].

As in Morley et al. [4], we divided the autosomal chromosomes into 491 windows of 5 Mb and determined the number of regulators mapping to each window. Table 1 presents the results for the five windows that have the most mapped phenotypes at a genome-wide significance level of 1.0 × 10^-5^. At the genome-wide significance level of 1.0 × 10^-3^, we found 68 hotspots with six or more hits, including the only 2 hotspots on chromosomes 14 and 20, with six and seven hits each detected by Morley et al. [4]. Even at the very stringent genome-wide significance level of 1.0 × 10^-5^, regulators for 51 phenotypes were found to map to the window on chromosome 10 (10p12).

**Table 1:** Number of mapped phenotypes at the genome-wide significance levels of 0.01, 0.05, 1.0 × 10^-3^, 1.0 × 10^-4^, and 1.0 × 10^-5^

		Count of mapped phenotypes^a^				
		
Chromosome	Region	*α = 0.05	α = 0.01	α = 1.0 × 10^-3^	α = 1.0 × 10^-4^	α = 1.0 × 10^-5^
10	26.35817Mb-29.46754Mb	244	117	61	56	51
19	60.12027Mb-63.63787Mb	52	25	17	17	17
21	46.14523Mb-46.84680Mb	91	34	18	15	15
22	21.40594Mb-24.67240Mb	79	34	25	23	23
22	42.36342Mb-43.48228Mb	54	19	15	15	15

^a^α, is the genome-wide significance level

**Figure 1:**
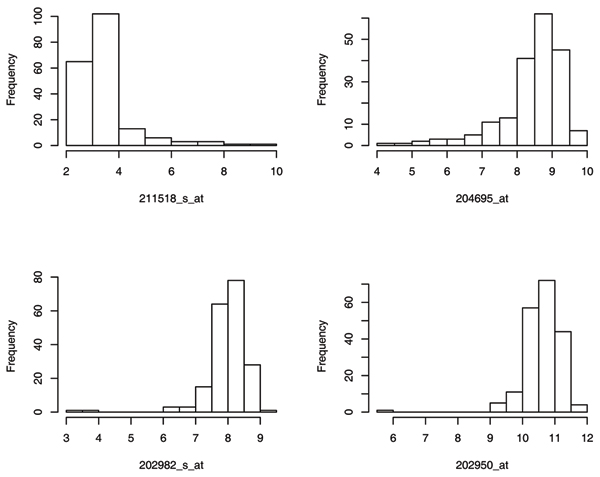
Histograms showing distributions of four non-normally distributed expression phenotypes.

Among the total 3554 expression phenotypes, almost half of them appear to be non-normally distributed by using the Shapiro test at a significance level of 0.05. At a significance level of 0.0001, the Shapiro tests for normality are still significant for more than 19% of phenotypes. Table 2 presents the summary statistics for four extremely non-normally distributed expression traits with *p*-values of Shapiro test less than 1.0 × 10^-12^: 211518_s_at, 204695_at, 202982_s_at, and 202950_at. The histograms of these four traits are shown in Figure 1. Phenotype 211518_s_at appears to be right-skewed and the other three appear to be left-skewed. There exist two and one outliers in the left for 202982_s_at and 202950_at, respectively, each with difference from the mean greater than seven times the corresponding standard deviation.

**Table 2:** Summary statistics of four non-normally distributed expression phenotypes

Gene	Mininum	Mean	SD	Maximum	Skewness	Kurtosis	*p*-Value of Shapiro test
211518_s_at	2.10	3.50	1.06	9.13	2.59	8.47	3.3 × 10^-17^
204695_at	4.21	8.44	0.94	9.79	-1.82	3.95	1.7 × 10^-13^
202982_s_at	3.20	7.98	0.68	9.19	-3.25	18.89	2.7 × 10^-16^
202950_at	5.99	10.62	0.59	11.80	-2.67	19.16	3.3 × 10^-13^

**Figure 2:**
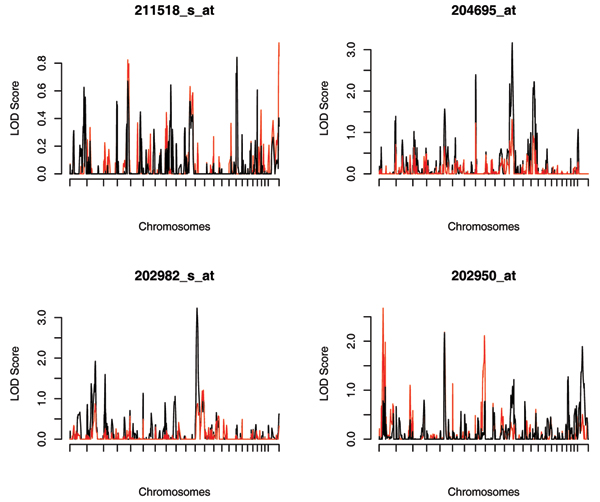
LOD score plots for four non-normally distributed expression phenotypes: SQTL (red solid) and standard VC approach (black solid).

We further performed multipoint genome-wide linkage analysis for the four non-normally distributed phenotypes and compared the performance of SQTL with that of the standard VC approach assuming normality. The multipoint IBD probabilities were estimated by using SOLAR. The whole genome linkage scan results are shown in Figure 2. For 211518_s_at, neither the SQTL nor the standard VC approaches detected any linkage signals. Linkage scans of 204695_at and 202982_s_at demonstrate that the standard VC approach is sensitive to non-normality and outliers and may obtain false-positive results. The standard VC approach fails to detect the eQTL on chromosome 1 identified by SQTL for 202950_at. This finding may reflect the fact that SQTL tends to be more powerful than the standard VC QTL mapping method in the presence of non-normally distributed data or outliers.

## Discussion

In this work, we applied the robust and powerful SQTL method to gene expression data and applied an efficient Monte Carlo procedure to account for multiple testing and to assess the genome-wide significance level. With the application to the GAW15 data, we detected many more eQTLs and hotspots at stringent genome-wide significance levels than Morley et al. [4]. Our new findings may be attributable to four factors. First, Morley et al. utilized only the data of siblings and much information from the parents and grandparents were missing, whereas the SQTL is applicable to arbitrary pedigrees. Second, in the context of VC analysis, SQTL tends to be more powerful than the regression-based methods including the one used in Morley et al.; see Diao and Lin [3]. Third, the SQTL approach is rank-based and tends to be more powerful than its parametric counterpart in the presence of nonnormal traits or outliers. Finally, the procedure used in Morley et al. to adjust for multiple testing is overly conservative.

The Monte-Carlo approach described in this work adjusts for multiple testing across the genome. It would be desirable to develop an efficient procedure to account for multiplicities across the genome and multiplicities across gene expressions simultaneously. Furthermore, the Monte-Carlo approach requires a large sample size in order to accurately approximate the joint distribution of the test statistics. Previous simulation studies demonstrated that the Monte-Carlo approach performed well for sample size as small as 50 pedigrees (results not shown). Future research is needed in evaluating the performance of the Monte-Carlo approach in small sample size situations.

With the availability of the abundance of SNPs, it is desirable to perform genome-wide association analysis of expression quantitative traits following genome-wide linkage analysis. We are currently extending the semiparametric quantitative transmission-disequilibrium test [8] to the genome-wide association analysis of expression data.

## Competing interests

The author(s) declare that they have no competing interests.

## References

[B1] Almasy L, Blangero J (1998). Multipoint quantitative-trait linkage analysis in general pedigrees. Am J Hum Genet.

[B2] Abecasis GR, Cherny SS, Cookson WO, Cardon LR (2002). Merlin-rapid analysis of dense genetic maps using sparse gene flow trees. Nat Genet.

[B3] Diao G, Lin DY (2005). A powerful and robust method for mapping quantitative trait loci in general pedigrees. Am J Hum Genet.

[B4] Morley M, Molony CM, Weber TM, Devlin JL, Ewens KG, Spielman RS, Cheung VG (2004). Genetic analysis of genome-wide variation in human gene expression. Nature.

[B5] Carlborg O, De Koning DJ, Manly KF, Chesler E, Williams RW, Haley CS (2005). Methodological aspects of the genetic dissection of gene expression. Bioinformatics.

[B6] Lin DY, Zou F (2004). Assessing genomewide statistical significance in linkage studies. Genet Epidemiol.

[B7] Lin DY (2005). An efficient Monte Carlo approaches to assessing statistical significance in genomic studies. Bioinformatics.

[B8] Diao G, Lin DY (2006). Improving the power of association tests for quantitative traits in family studies. Genet Epidemiol.

